# Evaluation of the Anti-Cancer Potential of Extracellular Vesicles Derived from Human Amniotic Fluid Stem Cells: Focus on Effective miRNAs in the Treatment of Melanoma Progression

**DOI:** 10.3390/ijms252312502

**Published:** 2024-11-21

**Authors:** Martina Gatti, Francesca Beretti, Gloria Ravegnini, Francesca Gorini, Eleonora Ceneri, Emma Bertucci, Matilde Y. Follo, Tullia Maraldi

**Affiliations:** 1 Department of Biomedical, Metabolic and Neural Sciences, University of Modena and Reggio Emilia, 41124 Modena, Italy; martina.gatti@unimore.it (M.G.); francesca.beretti@unimore.it (F.B.); 2 Department of Biomedical and Neuromotor Science, Cellular Signalling Laboratory, University of Bologna, 40126 Bologna, Italy; eleonora.ceneri@unibo.it (E.C.); matilde.follo@unibo.it (M.Y.F.); 3 Department of Pharmacy and Biotechnology (FABIT), University of Bologna, 40126 Bologna, Italy; gloria.ravegnini@unibo.it (G.R.); francesca.gorini@unibo.it (F.G.); 4 Department of Medical and Surgical Sciences for Mothers, Children and Adults, University of Modena and Reggio Emilia, 41124 Modena, Italy; emma.bertucci@unimore.it

**Keywords:** stem cells, extracellular vesicles, cancer, miRNA, redox balance

## Abstract

Mesenchymal stromal cells (MSCs) and their secretome show intrinsic antitumor properties, however, the anti-cancer effects of MSCs remain debated and depend on the cancer type or model. MSCs derived from discarded samples, such as human amniotic fluid (hAFSC), have been introduced as an attractive and potent stem cell source for clinical applications due to their collection procedures, which minimize ethical issues. Until now, various studies have obtained controversial results and poor understanding of the mechanisms behind the effects of perinatal cells on cancer cells. To better clarify this aspect, protein and miRNA expression profiling isolated from Extracellular vesicles (EVs) secreted by hAFSCs, obtained in the II or III trimester, were evaluated. Bioinformatic analysis was performed aiming at evaluating differential expression, pathway enrichment, and miRNA-mRNA networks. We highlighted that most of the highest expressed proteins and miRNAs are mainly involved in antioxidant and anti-cancer effects. Indeed, in the presence of hAFSC-EVs, a reduction of the G2/M phase was observed on melanoma cell lines, an activation of the apoptotic pathway occurred and the migration and invasion ability reduced. Our data demonstrated that II or III trimester hAFSCs can release bioactive factors into EVs, causing an efficient anti-cancer effect inhibiting melanoma progression.

## 1. Introduction

Several experimental studies have highlighted mesenchymal stromal cells (MSCs) as promising tools in cancer therapies [1] with the purpose of identifying new approaches that have fewer side effects but maintain efficacy in combatting one of the most important causes of mortality worldwide. MSCs can be obtained from the human body even from discarded tissues, such the perinatal ones, and can modulate angiogenic, proliferative, and apoptotic processes, leading to antitumor properties due to an intrinsic tropism toward malignant cells [2]. However, this effect is not fully proven, since MSCs exert the anti-cancer influence differently depending on the experimental condition such as the cancer cell type [3]. Indeed, MSCs can play opposing roles in the modulation of several pathways: production of immunosuppressive factors, involvement in the inhibition of antitumor immune response, and production of pro-angiogenic factors may support the tumor progression [4].

MSCs derived from perinatal tissues are highly suitable alternatives to bone marrow. Bone marrow-derived mesenchymal stem/stromal cells (BMSC) or adipose-MSCs can be obtained through easy and safe procedures routinely performed for other clinical purposes. They are even more interesting due to their young origin as they represent a more developmentally immature and juvenile cell source than adult somatic cells, while also being non-tumorigenic [5]. 

Various studies have investigated the antitumor potential of stem cells derived from human umbilical cord tissues, placenta, amniotic fluid, or membrane (hAFSCs and hAMSCs), however conflicting results were published [6,7]. Most of the studies have been conducted with hAMSCs and umbilical cord cells reporting a role in the promotion of vascularization, upregulating Akt/mTOR signaling, and promotion of metastasis. On the other hand, the secretion of mediators contrasting inflammation, migration, and inducing a cell cycle arrest have been demonstrated [1].

Looking at hAFSCs—the cell type studied in our lab for a decade for its regenerative potential—several independent studies have reported that hAF-MSCs and hAFSCs exert pro-survival, anti-apoptotic effects [8], while quenching inflammation [9] and stimulating local angiogenesis [10] in pathological models of tissue injuries. Sedrakyan et al. [11] showed in 2017 that these cells can modulate the VEGF cascade promoting angiogenesis, and Rosner et al. [12] provided evidence that hAFSCs can induce invasion of host cells causing pathological consequences and this stem cell-mediated invasion depends on aberrant induction of mTOR, HIF-1α, or MMPs. The opposite results have been reported even for hAFSC application: indeed, hAFSCs attenuated pancreatic cancer cell proliferation [13], ovarian cancer cell [14], and breast cancer cell line [15] viability.

The anti-cancer potential of perinatal cells has been investigated also in preclinical animal models, mostly on breast, colon, ovarian, and lung cancers [6]. In vivo studies on melanoma have been carried out with placenta, Wharton jelly, and amniotic epithelial membrane [6], but not yet with hAFSCs. 

Melanoma is responsible for the majority of skin cancer-related fatalities. BRAF mutations occur in about 50% of melanoma patients. FDA-approved BRAF and MEK inhibitors have improved the prognosis of BRAF-mutated patients, however, all responders develop resistance typically within one year of treatment [16]. Therefore, new treatments or co-treatments are urgently required in order to slow down the processes crucial for melanoma development, such as proliferation, survival, motility, and invasiveness, including distant metastatic niche formation.

In this study, c-kit^+^ hAFSCs were obtained from leftover samples of II trimester prenatal amniocentesis (fetal hAFSCs) and from clinical waste III trimester amniotic fluid during scheduled C-section procedures (perinatal hAFSCs).

Here, we tested in vitro the efficacy of extracellular vesicles (EVs) secreted by hAFSCs on the tumor progression signs of human melanoma cell lines (SK-MEL-28 and SK-MEL-2), harboring the BRAF600E and N-RasQ61R mutation, respectively. The use of EVs, instead of the cells, could be an easier but still effective therapeutic approach also in light of their putative low immunogenicity: moreover, even if the EVs contain several bioactive molecules, this system is much simpler than the whole cell. Also, in terms of investigating the mechanisms underpinned its efficacy. Indeed, proteins, mRNAs, and mostly miRNAs are considered the EV molecules able to regulate target cells [17]. Based on this consideration, we focused our attention on the most expressed miRNAs into hAFSC-EVs and their potential in counteracting cancer progression.

## 2. Results

### 2.1. Characterization of Cell Population from Amniotic Fluid of Second and Third Trimester

Amniotic fluids were collected from 6 amniocenteses, performed between the 16th and the 17th week, and 6 Cesarean procedures, performed between the 38th and the 39th week of gestation. No statistically relevant difference was appreciated in donor age between fetal II trimester (Amnio cells) and perinatal III trimester amniotic fluid (Cesarean cells) samples (mother age 37.7 ± 4.3 and 35.6 ± 3.7, respectively). 

Second-trimester amniotic fluid cells, obtained from the cell culture established for cytogenetic analysis purposes, represent a heterogeneous population and only 1% of them demonstrate stem cell characteristics: this sub-population was isolated with magnetic cell sorting for c-kit (CD117).

Unlike in mid-term, where isolation also involves c-kit sorting, most isolation of hAFSCs from full-term AF is performed using the straight-forward protocol in which the primary AF cells are cultured in a standard culture medium. This is probably due to the observation that, comparing cells obtained in the second trimester but at early (E) or late (L) weeks of pregnancy, the percentage of c-kit positive cells was 5 times higher in cultured E-AF, compared to cultured L-AF [18], indicating that this subpopulation (c-kit positive) is destined to decrease during the gestation, thus it would be not useful to isolate a so scarce subpopulation. Adherent cells derived from fresh AF samples from the third trimester in primary culture resulted in a mixed population with spindle and epithelioid-shaped morphology forming colonies at about 15 days of culture. Similarly, AF samples from the second trimester were cultured for 15 days before the sorting procedure.

**Figure 1:**
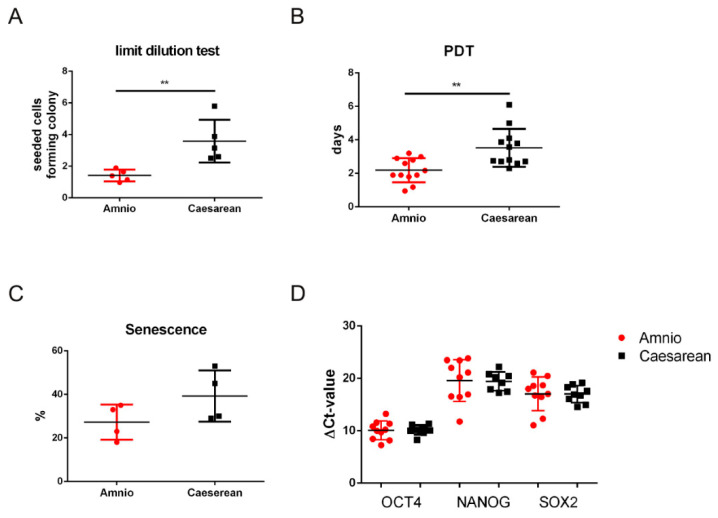
Comparison between hAFSCs derived from the Amnio and the Cesarean procedures. (**A**) Limit dilution test: graph showing the number of cells needed to form colonies. Each bar represents the 5 independent experiments obtained from 5 donors. Data were analyzed by *t*-test. ** *p* < 0.01. (**B**) Population doubling test (PDT) was performed as reported in the Methods section: the graph shows the comparison at the 4th passage in culture of 6 samples with two replicates for each group. Data were analyzed by *t*-test. ** *p* < 0.01. (**C**) Representative graph of β-galactosidase assay performed in 4 different Amnio and Cesarean samples at the 10th passage in culture. The number of senescent cells is expressed in percentage. (**D**) OCT4, NANOG, and SOX2 gene expression were analyzed. Triplicate reactions were performed for each experiment. Each bar represents the mean ± SEM of 3 independent experiments. Data were analyzed by *t*-test.

Comparing the proliferation capabilities, the dilution limit test highlighted a better performance for Amnio cells (Figure 1A), since the number of seeded cells able to form colonies is lower in Amnio cells samples. The growth rate of Amnio cells, measured as PDT at passage 4, is significantly higher than the one of Cesarean cells (Figure 1B). Both Amnio and Cesarean cells can be maintained in vitro culture for long periods, but different percentage of senescence was observed at passage 10, as from senescence-associated βgalatosidase activation: about 27% of Amnio cells and 42% of Cesarean cells (Figure 1C). 

RT-PCR was performed to test the expression of stemness markers, such as the transcription factors OCT4, NANOG, and SOX2. Although there was high variability, dependent on the donor, the mRNA levels were similar for both groups (Figure 1D).

**Figure 2:**
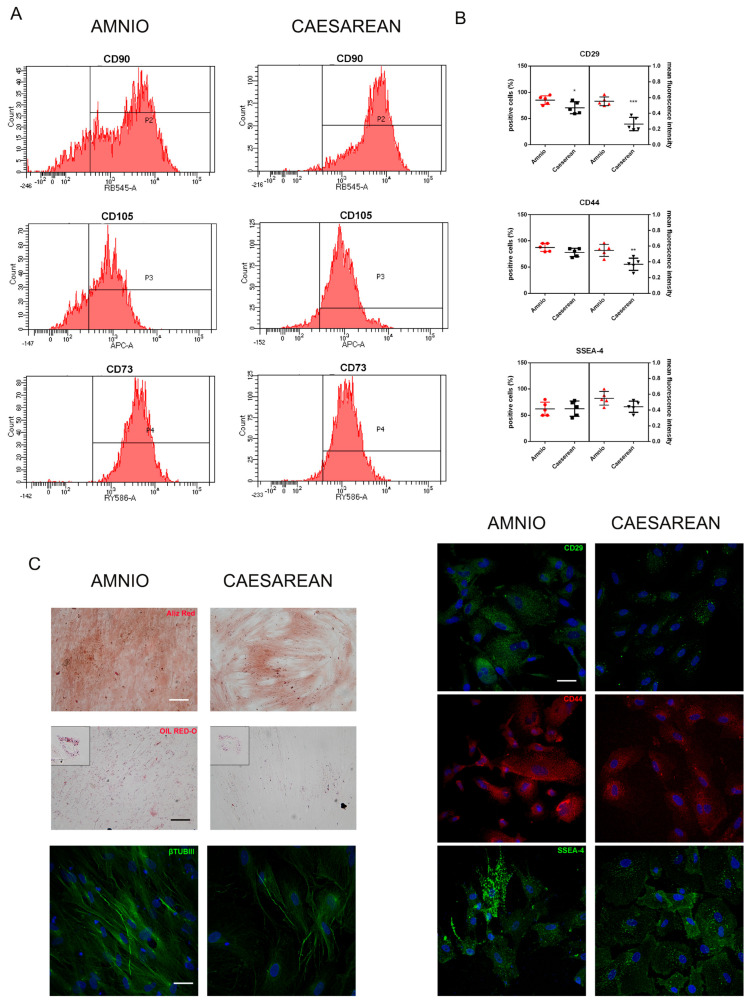
Comparison of the mesenchymal characteristics of hAFSCs derived from the Amnio and the Cesarean procedures. (**A**) Representative images of FACS analysis of the typical mesenchymal markers CD90, CD105, and CD73. The analysis was performed in three samples for each group. (**B**) Representative immunofluorescence images showing the expression of CD29, CD44, and SSEA-4. Images are superimposed between DAPI (blue) Scale bar = 10 µm. The images were analyzed for the number of positive cells and for the fluorescence intensity: the graphs on the left show the combined analysis for each marker. At least 100 cells were evaluated and t-test was performed. *** *p* < 0.001; ** *p* < 0.05; * *p* < 0.01 indicates samples significantly different. (**C**) Differentiation capability of Amnio and the Cesarean cells into the osteo, adipo, and neuro lineage. Differentiation after three weeks of exposure to osteogenic, adipogenic, or neurogenic medium is described in the Methods section. Evaluation of calcium deposition in the extracellular matrix through Alizarin Red staining: the intensity of red staining is related to the calcium presence typical of mineralizing tissue. Scale bar = 100 µm. Oil Red-O staining shows the presence of cytosolic lipid drops, related to adipogenic differentiation. The magnification of one cell is on the top left.—Representative confocal images of hAFSCs, labeled with DAPI (blue) and anti- β-TubulinIII (βTUBIII in green). Scale bar = 20 µm.

FACS analysis was performed to evaluate the percentage of positive cells for the three typical mesenchymal markers in order to define multipotent mesenchymal stromal cells. All the samples of Amnio and Cesarean cells, showed more than 80% of positive cells for the three markers, confirming the high presence of mesenchymal stromal cells. The fluorescence intensity is lower in Cesarean cells for CD73 and CD90. We tested also the positivity for the expression of CD45, CD14, and HLA-DR surface molecules. All markers were negative as expected (Amnio: CD45 = 0.9%, CD14 = 0.0%; HLA-DR = 0.0%. Caesarean: CD45 = 1.4%, CD14 = 0.0%; HLA-DR = 0.0%). The expression of the stage-specific embryonic antigen SSEA-4 was investigated using immunofluorescence (Figure 2B) since it is a surface glycosphingolipid. A slight difference between the two hAFSCs groups can be noticed when looking at the fluorescence intensity, not at the percentage of positive cells. This difference can explain the lower clonogenic capability and what we observed in the characteristics of cell surface mesenchymal markers of hAFSCs, determined by immunofluorescence as well (Figure 2A,B). Both Amnio and Cesarean cells expressed all the mesenchymal markers at high levels, considering the cell percentage. However, the results showed that MSCs from Cesarean cell samples showed lower intensity for the surface molecules CD44, CD73, CD90, and CD29.

Then, we analyzed the differentiation potential of hAFSCs. Furthermore, hAFSCs obtained from amniocentesis samples of different gestational times were analyzed for their capacity to differentiate between adipogenic, osteogenic, and neurogenic lineages. All clones of hAFSCs from amniocentesis samples and of the third trimester that grew in culture beyond 4 or 8 passages were able to differentiate in all lineages tested (Figure 2C). With osteogenic medium for 21 days, most cells exhibited extracellular matrix mineralization detected by Alizarin Red S staining. Similarly, after culturing with an adipogenic condition for 21 days, cells accumulated lipid vacuoles and exhibited intense staining with Oil Red-O. 21 days after neural induction, morphologically neural-like cells were observed by light microscope and a β-TubulinIII staining appeared. All the differentiation positivities were less intense for Cesarean cell samples.

### 2.2. EVs Isolation and Characterization

**Figure 3:**
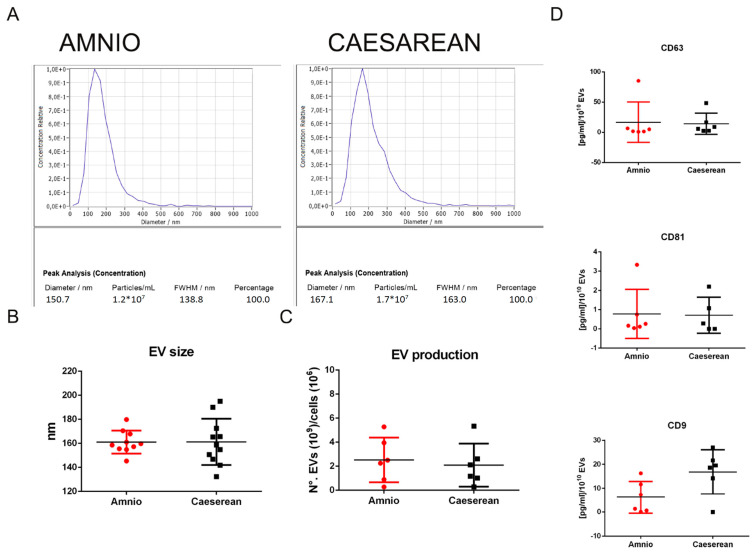
Analysis of the extracellular vesicles produced by Amnio and Cesarean cells. (**A**) Representative nanoparticle tracking analysis (NTA) performed on EV suspension with ZetaView. The mean diameter value and the number of particles of the presented analyses are at the bottom. (**B**) Graph representing the average of the mean diameter values obtained for 10 samples (EVs from CM obtained twice from each of the 5 donors) for each group. (**C**) Graph representing the average of the yields of extracellular vesicles obtained from Amnio and Cesarean fluid cells in the same experimental conditions. (**D**) ELISA analysis for CD63, CD81, and CD9 of the two groups of EV lysates.

hAFSC-CM samples were processed by concentration and ultracentrifugation to obtain EVs; both EVs showed an average size of around 160 nm measured by NTA (Figure 3A,B). EV yield was 2.53 ± 0.76 × 10^9^ particles and 2.09 ± 0.73 × 10^9^ for Amnio and Cesarean fluid 10^10^ cells, respectively (Figure 3C). hAFSC-EVs were found positive for the CD9, CD63 and CD81 tetraspanin signature (Figure 3D). Expression of these markers and of other EV typical proteins, such as Syntenin-1 and TSG101, were further confirmed by proteomic analysis.

**Figure 4:**
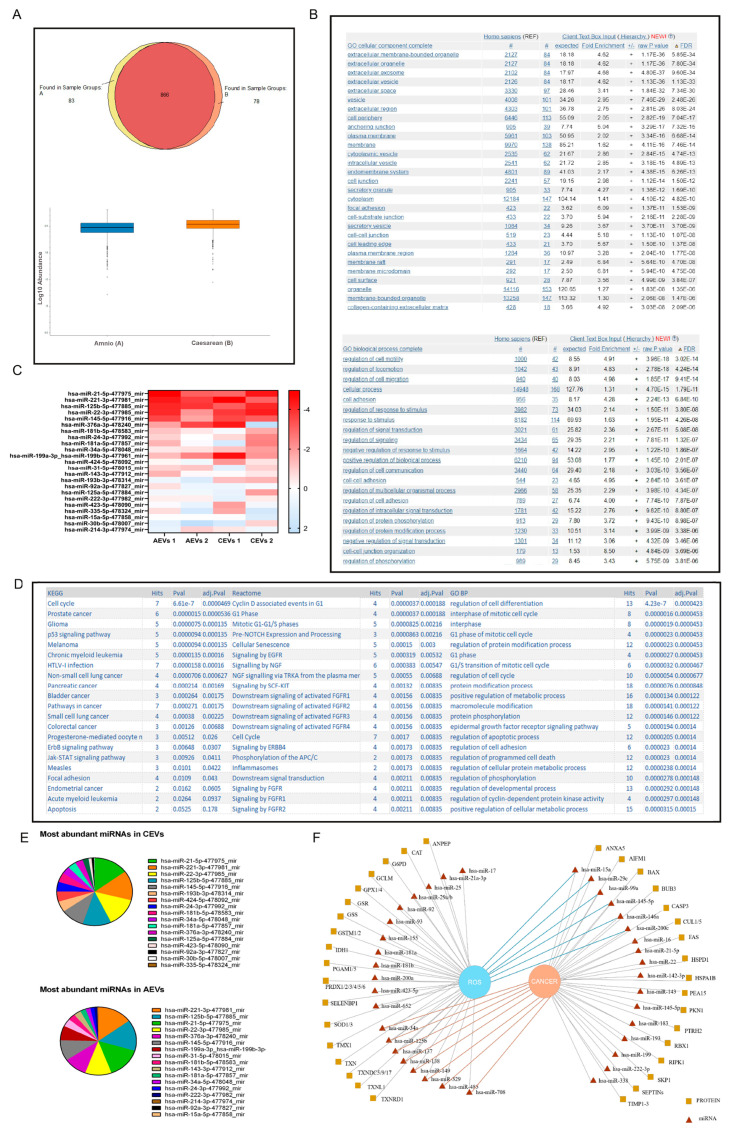
Proteome and miRNome analysis of the extracellular vesicles produced by Amnio and Cesarean cells. (**A**) Qualitative comparison of the EV protein content: group A represents the Amnio samples, group B the Cesarean ones. A total of 83 proteins are unique for A, and 78 for B, while 866 are in common. The bar graph represents the quantity of proteins present in each group. (**B**) Tables derived from Gene ontology (GO) analysis of cellular components, and biological processes, all shown with increasing FDR values. (**C**) Heatmap of the most expressed miRNA (23) in the two groups (AEVs and CEVs). hsa-miR16-5p was used as an endogenous control for data normalization. (**D**) Tables derived from KEGG, Reactome, and Gene ontology (GO) analysis of biological processes, all shown with increasing *p* values. (**E**) Pie charts showing the most abundant miRNAs in CEVs and AEVs. (**F**) Scheme illustrating the biological processes (decrease in ROS content and cancer pathways) in which EV- proteins and miRNA are involved, analyzing the literature.

The identified proteins, with high stringency (protein FDR confidence), within the hAFSC-EV cargo were 3028. Biological process and cellular component gene ontology (GO) analyses of the 1027 proteins with Abundance Ratio Variabilities of less than 30% suggested the presence of proteins involved in extracellular vesicle organization and transport (Figure 4B), confirming EV identity. The major part of these proteins (n = 866) was shared by the two groups and the abundance protein analysis showed similar levels for EVs obtained from the II and III trimesters (Figure 4A). These data suggest a similar protein pattern for Amniotic EVs (AEVs) and Cesarean EVs (CEVs), even if a minor part of proteins (n = 161) are unique for the group. Proteins expressed in all the samples with Abundances (Grouped) CV more than 30% in all the groups are 242. Comparing this list with the Vesiclepedia list [19], 27 proteins resulted in both the lists.

miRNA analysis of hAFSC-EVs, performed on around 400 miRNAs, showed that 67 of them were not expressed, while more than 320 were expressed in both the groups: among the highly expressed, the top 23 miRNAs are detailed in the list on the left of the heatmap. The top 5 expressed miRNAs are the same in both the groups (Figure 4E). Interestingly, GO enrichment analysis for biological processes highlighted the presence of miRNA species involved in anti-cancer processes, such as apoptosis and cell proliferation, and regulation of redox balance (Figure 4D). Reactome and KEGG analyses confirmed these data suggesting the presence of miRNA involved in the tumor progression. 

Then, we evaluated a wider list of the identified proteins and miRNAs present in the two groups: proteins with an Abundance Ratio between 0.5 and 2.0 and miRNAs whose ΔCt was lower than 4.0 in both groups were considered. Analyzing the literature for highly expressed proteins and miRNAs [20,21,22,23,24], we also appreciated that both can be related to several pathways involved in cell cycle/apoptosis, redox balance, and EMT, thus suggesting an EV protective role against tumor progression (Figure 4F).

### 2.3. hAFSCs-EVs Inhibited the Proliferation and Cancer Progression of SK-MEL-28 Cells

**Figure 5:**
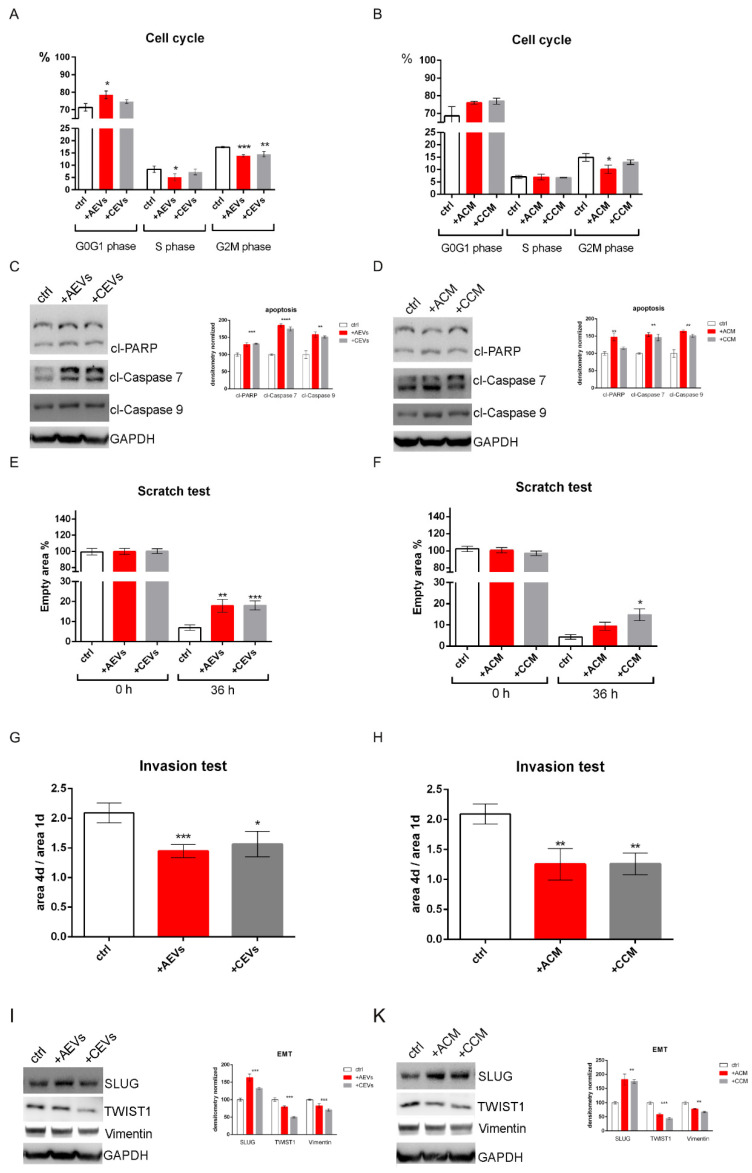
Effect of hAFSC secretome on melanoma cells. (**A**,**B**) Quantitative cell cycle analysis by flow cytometry of SK-MEL-28 treated or not with CM (**B**) or the derived EVs (**A**) obtained from Amnio or Caesarean cells samples. At least 10,000 cells were evaluated for each cytofluorimetric analysis. *** *p* < 0.001; ** *p* < 0.01; * *p* < 0.05 indicates samples significantly different. (**C**,**D**) Apoptosis induction: representative images of western blot analysis of SK-MEL-28 treated or not with CM (**D**) or the derived EVs (**C**) obtained from Amnio or Cesarean cells samples. Graphs represent the mean densitometric analysis of 3 independent experiments: cleaved PARP normalized full-length PARP, and cleaved caspases to GAPDH values. ANOVA test was applied. **** *p* < 0.0001; *** *p* < 0.001; ** *p* < 0.05 indicates samples significantly different. (**E**,**F**) The same samples were processed for migration analysis with a scratch test. The graphs show the area not invaded by cells, after 36 h. *** *p* < 0.001; ** *p* < 0.01; * *p* value < 0.05 indicates samples significantly different. (**G**,**H**) Graphs representing invasion assay results are shown as the ratio between the area reached after 4 days (4d) versus the one observed after 1 day (1d). *** *p* < 0.001; ** *p* < 0.01; * *p* value < 0.05 indicates samples significantly different. (**I**,**K**) EMT modulation: representative images of Western blot analysis of SK-MEL-28 treated or not with CM (**H**) or the derived EVs (**G**) obtained from Amnio or Cesarean cells samples. Graphs represent the mean densitometric analysis of 3 independent experiments normalized to GAPDH values. ANOVA test was applied. *** *p* < 0.001; ** *p* < 0.01 indicates samples significantly different.

To explore the growth inhibitory effects of hAFSC-EVs on SK-MEL-28 cells, the cell cycle analysis of SK-MEL-28 cells treated with both hAFSC-EVs for 72 h was evaluated. We demonstrated that hAFSC-EVs can arrest the cell cycle at the G1 phase of SK-MEL-28 cells (Figure 5A), even if not in a significant manner for all the parameters. Flow cytometry was conducted to identify changes in the cell cycle of SK-MEL-28 cells also treated with hAFSC-CMs (containing the EVs part) for 72 h. The result showed that hAFSC-CM-treated SK-MEL-28 cells underwent a G1 cell cycle arrest, due to the G2-M phase decrease (Figure 5B). This trend is similar to the one observed with hAFSC-EVs, but the statistical analysis showed a less interesting result. In general, there was no statistical difference between the AEVs and CEVs groups or between the Amnio CM (ACM) and Cesarean CM (CCM) groups.

The effects of hAFSC-EVs and hAFSC-CMs on apoptosis of confluent cultures of SK-MEL-28 cells were investigated by Western blot. The results showed that hAFSC-secretome treatment led to an increase in apoptotic cells of SK-MEL-28 cells (Figure 5C,D). The levels of pro-apoptotic factors such as PARP and caspase 7 and 9 in their cleaved forms were upregulated. Yet there was no apparent difference between these protein levels between hAFSC-EVs treatments and also in hAFSC-CM groups. Based on these data, hAFSC-EVs, included in a conditioned medium, could induce SK-MEL-28 cell apoptosis. The results obtained for apoptosis induction on another melanoma cell line SK-MEL-2, expressing mutant N-Ras (Q61R) and wildtype BRAF [25] showed a similar trend to the one observed for SK-MEL-28 (Supplementary Figure S1A). 

The migration of SK-MEL-28 cells treated with hAFSC-EVs and hAFSC-CMs was next assessed by performing scratch wound assays. Compared with the control group, hAFSC-EVs induced a significant decrease in SK-MEL-28 cell migration after 36 h, with a reduction of about half in their migration distance for both AEVs and CEVs (Figure 5E). A similar trend was observed in samples treated with hAFSC-CMs, however, in this condition, CCM seems to be more effective than ACM (Figure 5F). The migration capability of SK-MEL-2 was less compared to the one of SK-MEL-28, but the trend of the treatment effect was similar (Supplementary Figure S1B).

To test the effect of hAFSC-secretome on the capability of SK-MEL-28 cells to invade, 3D invasion assays using spheroids embedded in matrigel were performed. In our previous study on SK-MEL-28, we observed that cells induced to be resistant to BRAF inhibitor (R) expressed higher levels of ZEB1 and lower of Slug, compared to sensitive cells (S), supporting their higher invasive profile due to the phenotype switching. For this reason, we performed an EMT test and invasion assay with R cells. The result showed that compared with the control group, both hAFSC-EVs and hAFSC-CMs treatments impaired in a similar manner, SK-MEL-28 cell invasion, as shown by the significant reduction of about 30–40% in the area of spheroids of invading cells (Figure 5G,H).

Owing to the pivotal role the EMT pathway plays in melanoma development and progression, whether hAFSCs-EVs and hAFSC-CMs could affect this signaling pathway was examined. SK-MEL-28 cells with or without treatment of hAFSC-secretome were used to analyze the expression of SLUG, TWIST1, and vimentin proteins. SLUG was upregulated unlike vimentin and TWIST1 (Figure 5I,K). These effects were confirmed in SK-MEL-2 cells (Supplementary Figure S1C) and were related to an inhibition of SK-MEL-28 migration, as shown in Figure 5E,F, and a reduction in invasion capability (Figure 5G,H).

All these data suggest that CMs and the derived EVs exert comparable effects on melanoma cells. Notably, CMs and EVs obtained from amniotic cells of different gestational ages are similar in the more abundant protein and miRNA content.

## 3. Discussion

MSC-EVs are crucial paracrine regulators in a number of physiological and pathological processes [26]. Thanks to a strong tendency to reach injuries and tumor sites in vivo, MSC-EVs hold great promise as cell-free therapy for a variety of diseases [27,28]. However, the exact effects of MSCs and of MSC-EVs on tumor development and progression remain controversial [29]. Some studies have shown promoting effects, while others have demonstrated inhibitory effects of MSC-EVs on tumors [30,31]. These different results could be attributed to the different tissue origins of MSCs and tumor types. The amniotic fluid is a pre- or neonatal-related sample, usually discarded, in which MSCs are abundant. The amniocentesis procedure is now less common since it was replaced by other less invasive approaches for prenatal cytogenetic investigations. It is crucial to prove the therapeutic efficacy of amniotic fluid cells obtained at term, during Cesarean delivery. For this purpose, we compared the Amnio and the Cesarean cells, starting from the stem cells and EVs characteristics, showing similarities and differences. Then, we revealed that both human amniotic fluid-derived MSC-EVs had strong antitumor effects on melanoma cells: hAFSC-EVs decreased the proliferation, migration, and invasion of cultured SK-MEL-28 cells and induced cell apoptosis.

Here we showed that amniotic cells obtained from the second and the third trimester express stem cell transcription factors in a similar manner: OCT4 seems to be the most expressed as mRNA, compared to SOX2 and NANOG. Moreover, we analyzed another embryonic marker, SSEA-4, since it has been reported that AF-MSCs mainly expressed SSEA-4 with very weak expression of OCT4, SOX2, and NANOG [32]. SSEA-4, revealed as protein presence, is expressed in more than 50% of cells, even with a lower level in Cesarean cells. The best proliferation and delayed senescence of Amnio cells may partially coordinate its high expression of the pluripotent markers SSEA-4 and c-kit. Indeed, Amnio cells were selected for c-kit, as often reported in the literature [33]. The percentage of c-kit cells is around 1% in the second trimester and decreases with the gestational age [18], so it is useful to isolate this subpopulation, and then expand it in culture, only for samples deriving from amniocentesis. It was recently demonstrated that analyzing clones present in amniotic fluid cell populations in the second trimester, the one expressing c-kit (20%) showed higher CD90, CD105, and CD117 levels, correlating with increased proliferation capacity. Minimal criteria established by the International Society for Cellular Therapy (ISCT) defined MSCs only for the expression of the CD105, CD73, and CD90 markers. However, MSCs can be characterized by CD44 and CD29 expression. CD90, a glycoprotein expressed on the MSC membranes, is related to the state of cellular differentiation and might be related to the growth rate. CD44 is a cell surface receptor, considered one of the most important adhesion molecules. CD90 has a direct relationship with CD44 since a decrease in CD90 expression reduces CD44 expression and increases the cell differentiation state, suggesting that CD44 associated with CD90 may also influence the stemness state of MSCs. Of note, the expression levels of “stemness” markers (CD44 and CD105) were higher in MSC populations with more characteristic mesenchymal stem cell morphology and phenotypes [34]. Based on these observations, it is not surprising to observe a better differentiation capability for Amnio cells than Cesarean cells.

The paracrine effect of MSCs can be exerted by the secretome and the contained EVs. These vesicles are largely studied because of their easy therapeutic applicability. The EVs biocargo, including DNA, RNA, miRNAs, proteins, and other molecules, is abundant. Comparing EVs obtained from Amnio and Cesarean secretome, relevant differences cannot be noticed, not regarding the size, the yield, or the expression of exosome markers. The protein content is similar for the majority of the identified proteins and demonstrates the identity of the source, namely extracellular vesicles. The role of these proteins is mostly included in cell adhesion and communication, but a clear suggestion on a possible regulating role of EVs on cancer was not derived from the proteome analysis. Indeed, many proteins with opposite effects on cell proliferation were identified. Notably, proteins clearly involved in the promotion of the apoptotic process were identified, such as AIFM1, caspase 3, BAX, and FAS, as reported in ApoCanD [21]. In the scheme shown in Figure 4F, other pro-apoptotic proteins and cell cycle regulating proteins were reported, as indicated by the Proteome Discoverer analysis in the classification by Wiki and Reactome Pathway. In our point of view, it was more interesting to note that many antioxidant proteins have been identified in both groups, and all of these are shown on the left side of Figure 4F. We have already demonstrated that SOD1 is highly represented in EVs derived from II trimester hAFSCs [35] and recently the Bollini group [36] showed the presence of several redox-modulating proteins in EVs from II trimester hAFSCs as well. Here, we demonstrated that this antioxidant potential can be obtained also from hAFSC-EVs of the III trimester. 

miRNA analysis revealed that the most expressed miRNA can regulate the cell cycle, p53 signaling pathway, and apoptotic process. Notably, the analyzed miRNAs are mostly present in both the EVs (from Amnio and Cesarean), supporting the similarity of these vesicles.

The miRNA list shows the most expressed miRNAs in AEVs and CEVs, having normalized the values on miR-16, widely used as housekeeping miRNA [37]. Indeed, we evaluated miR-16 in all our samples and observed that the values remained stable. However, miR-16 is a pivotal miRNA in melanoma, regulating the cell cycle and apoptosis. Consistent with previous studies, Guo et al. (2016) found that overexpressed miR-16 could markedly induce cell apoptosis, cell cycle arrest, and proliferation inhibition in melanoma cell lines. Moreover, they proved that miR-16 could suppress melanoma growth in a xenograft mouse model, contributing to melanoma progression [38,39]. 

In this list, many other miRNAs are reported as inhibitors of tumor growth. miR-21-5p is one of the most expressed but its role is often reported as a tumor inducer; however, in 2021 Du et al. showed that MSC-EVs dramatically inhibited migration and invasion behaviors in breast cancer through downregulation of ZNF367 and upregulation of miR-21-5p [40]. A review by Jahangiri et al. [41], on the effects of miRNAs contained in EVs derived from different sources of MSCs on cancer, described also miR-143, miR-193, miR-199, and miR-222-3p, which are present in our top 23 list as tumor suppressive in melanoma [22].

Notably, the top 5 miRNAs are the same in the two groups and three of them have a suppressing role in melanoma. When miR-22-3p is overexpressed in WM-266-4 melanoma cells, the cell viability is decreased, the expression levels of LGALS1, VIM, and SNAI2 are decreased, the expression level of CDH1 is increased, and cell apoptosis is increased [42]. The expression levels of miR-145-5p are decreased in melanoma tumor tissues and cell lines. SOX2 is a target of miR-145-5p since it is downregulated by this miRNA, and its expression promotes the proliferative, migratory, and invasive abilities of melanoma cells [43]. It is intriguing that miR-145-5p overexpression decreased superoxide anion levels. A decrease in miR-145-5p and an increase in NOX1, producing superoxide anion, were observed following ischemia/reperfusion injury [44]. MiR-125b has been found to be upregulated in some tumor types, e.g., colon cancer and hematopoietic tumors, where it displays an oncogenic potential, by inducing cell growth and proliferation and blocking apoptosis. In contrast, it acts in other tumor entities, e.g., melanoma, as a tumor suppressor by targeting c-Jun and promoting cell death [45]. Indeed, restoring miR-125b levels in melanoma cells shows promise as a treatment, and combining it with other therapies could improve effectiveness [22,46]. Moreover, activation of the ROS-producing enzyme isoforms NOX2 and NOX4 in neuronal cells (PC-12) was reversed by the inhibition of miR-125b [47].

Interestingly, many other highly expressed miRNAs can regulate redox balance, reduce oxidative stress, and exert a tumor-suppressing role. miR-15a is a crucial tumor suppressor in melanoma. It targets and reduces the expression of key genes involved in cell cycle progression and apoptosis, such as CCNE1 and BCL2 [48]. Additionally, miR-15a suppresses multiple oncogenes and weakens the tumor-suppressive environment when its levels decrease. Moreover, miR-15a demonstrated antioxidant properties by reducing the expression of NOX5, another ROS-producing enzyme [49]. 

Similarly, miRNA-708 can play a double role. For instance, its overexpression successfully reduced N-Ras protein levels in melanoma carrying this mutation [50] and decreased ROS levels in other contexts [51], even though it is still debated.

As a tumor suppressor, miR-146a targets genes involved in cell proliferation and survival, inhibiting oncogenes and promoting apoptosis. It also regulates the cell cycle; when downregulated, it can lead to uncontrolled cell proliferation and resistance to apoptosis. Furthermore, mir-146a inhibits metastasis by targeting genes involved in cell migration and invasion, such as MMPs, and influences the process of the epithelial–mesenchymal transition (EMT), thereby impacting the metastatic behavior of melanoma cells [52,53]. Also, miR-146a acts as a redox modulator since it can decrease the expression of NOX4 [54]. miR-34a, miR-137, miR-99a, and miR-21a-3p targeting NADPH oxidases are predominantly downregulated in ROS-driven cancers and are defined as onco-suppressors [55]. They are present in both hAFSC-EVs. In particular, miR-34a and miR-137 [56,57] inhibit melanoma cell migration and invasion.

The complexity of EV cargo is high and we are aware that several miRNAs and proteins identified as EVs have promoting roles in cancer, but the identification of miRNAs and proteins highly expressed with antioxidant and pro-apoptotic effects supports the role of EVs in redox balance thus leading to an anti-tumor outcome. 

Therefore, we performed in vitro analysis of the effects of EV exposure on melanoma cells. All the parameters investigated suggest that EVs (Amnio and Cesarean) efficiently reduced cancer growth and spread. These effects were the same when obtained from the conditioned media from which EVs were derived, confirming the crucial role of EVs in paracrine modulation by AFSCs. This study clearly shows the efficacy of this cell-free approach in melanoma cells, without a cargo artificial enrichment process. This efficacy was observed in melanoma cells carrying BRAF as well as N-Ras mutation, namely SK-MEL-28 and SK-MEL-2, suggesting that the ERK pathway is the point more crucial than the Akt pathway for the treatment with EVs (Amnio and Cesarean). Melanomas, like many other malignancies, show signs of increased oxidative stress both inside the cells themselves and in the surrounding microenvironment of the tumor. Even though innovative treatments for melanoma, such as targeted and immunological therapy, have improved patient survival, chemoresistance inhibits further improvements in patient survival. Oxidative stress and redox homeostasis are implicated in all phases of melanoma genesis as well as in the emergence of drug resistance. However, the role of oxidative stress is somewhat paradoxical: while ROS help to promote cancer survival, proliferation, tumor vascularization, and metastasis, at high levels, they can also cause DNA damage and cancer cell death [58]. Acquired resistance to BRAF/MEK inhibitors can lead to disease relapse, and this is thought to at least be in part due to redox metabolic rewiring [59]. Thus, treatment modalities that affect the oxidative stress pathways may prove especially beneficial in drug-resistant melanomas. Furthermore, a therapeutic approach combining the capacity to reduce cell proliferation, regulate redox balance, and affect EMT could be more effective and synergic. These in vitro data should be confirmed in in vivo experiments where the regulation of EVs on vascularization/angiogenesis could be evaluated. 

In conclusion, the similarity in EV content derived from second- and third-trimester hASFCs explains the analogous effects of hAFSC-EVs on melanoma cells. The anti-cancer effect of hAFSC-EVs may be exerted through the synergetic interaction of numerous active factors. 

## 4. Materials and Methods

### 4.1. Amniotic Fluid Stem Cell Isolation

hAFSC were isolated from leftover samples of amniotic fluid (AF) collected by routine prenatal screening via II trimester amniocentesis (Amnio cells), or as clinical waste during scheduled Cesarean-section delivery during the III trimester (Cesarean cells) at the Unit of Obstetrics & Gynecology, at the Policlinico Hospital of Modena (Italy), all with patient consent as well as institutional ethical approval (protocol 360/2017 dated 15 December 2017 approved by Area Vasta Emilia Nord).

Amnio hAFSCs were obtained from amniotic fluids collected from 6 healthy pregnant women at the 16th week of gestation who underwent amniocentesis due to maternal request (not for fetal anomalies). For this study, supernumerary (unused) flasks of AF cells, cultured in the Laboratory of Genetics of TEST Lab (Modena, Italy) for 2 weeks, were used. Amnio hAFSCs were isolated as previously described [30]. Human amniocentesis cultures were harvested by trypsinization and subjected to c-kit immunoselection by MACS Technology (Miltenyi Biotec, Bergisch Gladbach, Germany). hAFSCs were subcultured routinely at 1:3 dilution and not allowed to grow beyond 70% confluence in culture medium (αMEM) supplemented with 20% fetal bovine serum (FBS), 2 mM L-glutamine, 100 U/mL penicillin, and 100 μg/mL streptomycin (all from EuroClone Spa, Milano, Italy). 

Cesarean hAFSCs were obtained from 6 amniotic fluid samples from healthy human donors, collected during full-term C-sections. In general, the time between collection and processing was kept as short as possible to minimize cell death. First, cells were collected by gradient Ficoll separation, then washed with PBS and centrifuged at 300× *g* for 5 min. The supernatant was discarded, and the pellet was washed again with PBS and dissolved in Ammonium chloride to reach 0.8% to lyse the remaining erythrocytes. Thereafter, the cell solution was incubated at 4 °C for 20 min and centrifuged again. This procedure was repeated until the pellet had a clear color. Afterward, the cells were cultured in culture medium (αMEM), supplemented with 20% fetal bovine serum (FBS), 2 mM L-glutamine, 100 U/mL penicillin, and 100 μg/mL streptomycin (all from EuroClone Spa, Milano, Italy). Once attached, the cells were visible after 7–10 days and the medium was changed.

### 4.2. Limit Dilution Test

A limiting dilution assay was performed, plating the cells at 250, 125, 62.5, 31, 16, 8, 4, 2, and 1 cell per well in a 96-well plate to evaluate the clonogenic potential. After 10 days, cells were fixed with methanol/acetic acid 3:1 for 5 min and stained with 0.5% crystal violet in methanol for 30 min at room temperature. The number of colony-forming units (CFU) generated was counted as described in [60].

### 4.3. Cellular Proliferation

Cells were seeded in a T25 cm^2^ flask at a density of 2000 cells/cm^2^, cultured for 3,5 days then detached, counted, and seeded again at 2000 cells/cm^2^. Cultures were performed until passage 8 and population doubling time (PDT) for each passage was measured by applying the formula reported in [61].

### 4.4. Senescence Assay 

In order to evaluate the presence of senescent cells in fractions of hAFSCs, cells at passages 6–8 were seeded in 12-well plates and processed using a senescence β-galatosidase staining kit (Cell Signaling, Danvers, MA, USA), according to the manufacturer’s instructions.

### 4.5. FACS Analyses

Adherent cells were harvested for surface antigen analysis. Briefly, cells were detached from plastic support by trypsin (EuroClone), counted, and aliquoted in FACS analyses polypropylene tubes (0.5–1 × 10^6^ cells/tube) (VWR, Milan, Italy). EDT-MSCs were subsequently incubated in blocking buffer (100 μL each 0.5–1 × 10^6^ cells) containing Dulbecco’s Modified Eagle’s Medium (DMEM, Gibco from Sigma–Aldrich, St. Louis, MO, USA), 10% FBS (PAA Laboratories now GE Healthcare, Irvine, CA, USA), and 0.1 M sodium azide and human immunoglobulin G (both from Sigma) and incubated for 20′ on ice. After a PBS (PAA Laboratories) washing step, cells were resuspended in PBS (PAA Laboratories) with 0.5% bovine serum albumin (BSA, Sigma –Aldrich, St. Louis, MO, USA) and stained on ice and in the dark for 30′ with the following monoclonal antibodies: APC-anti-CD45, FITC-anti-HLADR, PE-anti-CD14, (all from Becton Dickinson, Franklin Lakes, NJ, USA); APC-anti-CD90, FITC-anti-CD105, PE-anti-CD73 (all from BD Pharmingen, Franklin Lakes, NJ, USA). In all the experiments, the corresponding isotype-matched antibodies were used as negative controls (BD Pharmingen and Becton Dickinson, Franklin Lakes, NJ, USA). Data were collected using a FACS Aria III flow cytometer (BD Biosciences) and analyzed on FACS Diva v. 9.0 software (BD, Franklin Lakes, NJ, USA). In all the experiments, the corresponding isotype-matched antibodies were used as negative controls (BD Pharmingen and Becton Dickinson, Franklin Lakes, NJ, USA). Data were collected using a FACS Aria III flow cytometer (BD Biosciences, Franklin Lakes, NJ, USA) and analyzed on FACS Diva software (BD Biosciences, Franklin Lakes, NJ, USA).

### 4.6. Immunofluorescence and Confocal Microscopy

For immunofluorescence analysis, hAFSCs were processed, and confocal imaging was performed using a Nikon A1 confocal laser scanning microscope, as previously described [33]. Primary antibodies to detect CD29/Integrinβ1 and CD44/HCAM (Santa Cruz Biotechnology, Biotechnology, CA, USA), CD105/Endoglin, CD90 (Millipore, Burlington, MA, USA), CD73 (Gene Tex, San Antonio, TX, USA), SSEA-4, and β-TubulinIII (Cell Signaling, Danvers, MA, USA), were used following datasheet recommended dilutions. Alexa secondary antibodies (Thermo Fisher Scientific, Waltham, MA, USA) were used at 1:200 dilution.

The confocal serial sections were processed with ImageJ-1.51 software to obtain three-dimensional projections. The image rendering was performed by Adobe Photoshop software. The cell fluorescence signal (mean fluorescence intensity) was quantified using ImageJ and applying the following formula: Integrated Density—(Area of selected cell X Mean fluorescence of background readings).

### 4.7. RNA Isolation and Quantification

RNA was isolated using TRIzol^®^ Reagent (Invitrogen, Waltham, MA, USA) following the manufacturer’s protocol. Starting from 1 μg of the extracted RNA, the cDNA was obtained using SensiFASTTM cDNA Synthesis Kit (Meridian Life Science Inc., Cincinnati, OH, USA) following the manufacturer’s protocol. Real-time PCR was performed using SsoAdvanced Universal SYBR Green Supermix following the manufacturer’s protocol (temperature). Real-time PCR reaction was carried out in a total volume of 10 μL loading 250 ng of cDNA and 500 nM of each primer. cDNA amplification was performed by activating the polymerase for 30 s at 95 °C, followed by 40 cycles of 5 s at 95 °C and 30 s at 60 °C. Normalized expression levels were calculated relative to control cells according to the ΔCT method. Primer sequences used in this study are listed below [62]:

- NANOG:Fw CCAGAACCAGAGAATGAAATC, Rv TGGTGGTAGGAAGAGTAAAG, (NM_024865);
- SOX2: Fw ATAATAACAATCATCGGCGG, Rv AAAAAGAGAGAGGCAAACTG, (NM_003106);
- OCT4: Fw AGAGAAAGCGAACCAGTATC, Rv TTACAGAACCACACTCGG, (NM_002701.5);
- GAPDH: Fw ACAGTTGCCATGTAGACC, Rv TTGAGCACAGGGTACTTTA, (NM_002046);

### 4.8. Differentiation Protocols

Osteogenic differentiation was obtained in a medium composed by aMEM supplemented with 10% FBS, 2 mM L-glutamine, 100 U/mL penicillin and 100 mg/mL streptomycin (all from EuroClone Spa, Milan, Italy), 100 mM 2P-ascorbic acid, 100 nM dexamethasone and 10 mM β-glycerophosphate (all from Sigma-Aldrich, St. Louis, MO, USA).

Neurogenic differentiation protocol: cells cultures (seed at 60% of confluence) were maintained in aMEM supplemented with 10% fetal bovine serum and 20 µM retinoic acid in dimethyl sulfoxide, (all from Sigma-Aldrich, St. Louis, MO, USA) [63].

Adipogenic differentiation protocol: cells were incubated in adipogenic induction medium composed of high glucose DMEM culture medium (Thermo Fisher Scientific, Vantaa, Finland) supplemented with 10% FBS (GE Healthcare, Buckinghamshire, UK), 1% penicillin/streptomycin (Lonza, Basel, Switzerland), isobutylmethylxanthine, 1 μM dexamethasone, 10 μg/mL insulin, 0.2 mM indomethacin (all from Sigma-Aldrich, St. Louis, MO, USA). After 3 days, the medium was replaced with one containing only 10% FBS, 2 mM L-glutamine, 100 U/mL penicillin and 100 μg/mL streptomycin, and 10 μg/mL insulin [62].

All differentiation media were changed twice a week and cells were kept in these conditions for at least 3 weeks in an incubator at 37 °C with 5% CO_2_.

### 4.9. Histological Staining

Fixed monolayer cells were washed with distilled water and then incubated with a 2% Alizarin Red S solution at pH 4.2 for 10 min at RT. Images of histological samples were obtained with a Zeiss Axiophot microscope (Zeiss AG, Jena, Germany), equipped with a Nikon DS-5Mc CCD color camera.

To evaluate adipogenic differentiation, cells were washed with 3% isopropanol (Sigma–Aldrich, St. Louis, MO, USA). Samples were stained with 0.5% (*w*/*v* in 60% isopropanol) Oil Red-O (Sigma–Aldrich, St. Louis, MO, USA) to detect cytoplasmatic lipid droplet accumulation and counterstained with hematoxylin.

### 4.10. EV Isolation

hAFSCs were grown in 75 cm^2^ flasks until sub-confluence (around 1 × 10^6^ cells). Before extracellular vesicle extraction, the cells were maintained for 4 days in a 10 mL culture medium deprived of FBS in order to exclude the contamination by extracellular vesicles included in the FBS solution. The secreted part of the conditioned medium (CM) was centrifuged at 300× *g* for 10 min at 4 °C and then concentrated up to 2 mL by using Centrifugal Filter Units with 3K cutoff [64]. Part of the concentrated CM was stored at −80 °C and used for treatment. Next, part of concentrated CM was again centrifuged at 10,000× *g* for 30 min at 4 °C and the supernatant was transferred to poly(propylene) ultracentrifuge tubes (13.2 mL, Beckman Coulter, Milano, Italy). The supernatant was centrifuged at 100,000× *g* for 90 min at 4 °C in a Beckman Coulter Optima L-90K centrifuge with a SW-41 rotor, and the supernatant was discarded while pellets were resuspended in 13 mL PBS and centrifuged again at 100,000× *g* for 90 min at 4 °C. The pellet of EV was resuspended into 100 μL of PBS for spectrophotometric and NTA analysis.

After dilution 1:1000, the size distribution and concentration of EVs were analyzed by nanoparticle tracking analysis using a ZetaView particle tracker from ParticleMetrix (Ammersee, Germany).

### 4.11. ELISA Assays

CD63, CD81, and CD9 were quantified in the lysed EV of hAFSCs by using ELISA assays, according to the manufacturer’s instructions (Cusabio, Huston, TX, USA). In brief, EVs were lysed in a lysis buffer at a ratio of 1:3 (*vol*:*vol*) followed by three cycles of freeze and thaw [65]. Protein concentration was found in all EV preparations, as analyzed by the Bradford test. Samples were run in duplicate. A standard curve was constructed using known concentrations of recombinant human standards.

### 4.12. SDS PAGE and Western Blot

Whole-cell lysates were processed as previously described [16]. Primary antibodies were raised against the following molecules: PARP, GAPDH, SLUG (Santa Cruz Biotechnology, Santa Cruz, CA, USA), cl-caspase-7, cl-caspase 9, vimentin (Cell Signaling Technology, Lieden, The Netherlands). Secondary antibodies, used at 1:3000 dilution, were all from Thermo Fisher Scientific (Waltham, MA, USA).

### 4.13. Mass Spectrometry and Bioinformatic Analysis

EVs from different sources were processed with a Filter Aided Sample Prep (FASP) protocol to obtain a quantitative protein extraction and high-yield generation of peptides [65]. Briefly, 30 µL of EV in PBS were lysed in 200 µL of Urea Buffer (8 M urea in 0.1 M Tris/HCl pH 8.5) by sonication, transferred to the filter device with 10 kDa membrane filter cut-off (Millipore) and then centrifuged at 14,000× *g* for 15 min.

The filter device supernatant underwent overnight protein digestion using trypsin enzyme 1:100 (*w*/*w*) in Ammonium bicarbonate solution (50 mM NH_4_HCO_3_ in water) after disulfide bridge reduction and alkylation by treatment with 8 mM DTT incubation and carbamide methylation by 50 mM iodoacetamide, respectively. Following digestion, the tryptic-digested fragments present in the supernatant were collected in the filtrate by centrifuging the filter units at 14,000× *g* for 10 min, acidified with 1% Trifluoracetic Acid, and dried up the sample using a Speed vacuum.

LC-MS/MS analysis was performed using Exploris 480 Hybrid Quadrupole-Orbitrap™ Mass Spectrometer (Thermo Fisher Scientific, USA) coupled with a Thermo Ultimate 3000 Nano UHPLC. Samples were analyzed in triplicates, using 1 µg per injection. Chromatographic separation was performed on a 75 µm × 500 mm Easy Nano C18 column (Thermo Scientific, Waltham, MA, USA). 

Raw MS files were analyzed with the Proteome Discoverer software (v. 3.0.1.27) from Thermo Fisher Scientific with a canonic LFQ (Label-Free Quantification) workflow. Protein ID was performed against the Human Uniprot database (NCBI: txid9606). The allowed peptide modifications were carbamidomethylation (C) (fixed) and oxidation (M) (variable), and the enzyme specificity was set to trypsin, with maximum missed cleavages set to 2. The precursor and fragment mass tolerances were set to 10 ppm and 0.02 Da, respectively. A search was run on both Sequest HT with Inferys rescoring and MSAmanda 2.0. The false discovery rate (FDR) of the peptide-to-spectrum matches was calculated by a Percolator and set to 0.01. Proteins were quantified using MS1 intensity. Protein identification against the peak list was performed in MASCOT version 2.1 with Swiss-Prot and cRAP database as the search engine. The search parameters for database search using Mascot were given as, taxonomy: human; enzyme used for digestion: trypsin with one missed cleavage allowed; fixed modification specified as carbamidomethylation (C), and oxidation (M) and deamidated (NQ) as variable modifications. The peptide mass tolerance was set as 40 ppm and 0.08 Da for MS/MS tolerance. The lists of identified proteins were subjected to the PANTHER classification system, version 9.0 (http://www.pantherdb.org/ URL (accessed on 8 May 2024), to understand the biological context of the identified proteins and their involvement in biological pathways. The list of UniProt Accession numbers was uploaded and mapped against the reference Homo sapiens dataset to extract and summarize molecular functions, biological processes, and the class of proteins. The above result was further processed in order to get the proteins of the top five categories for each of the functional domains.

### 4.14. miRNA Analysis

RNA was isolated from hAFSC-EVs using the RecoverAll Total Nucleic Acid Isolation Kit (Ambion, Thermo Scientific, Waltham, MA, USA) following the manufacturer’s instructions. RNA integrity and quantification were evaluated using the 2100 Agilent Bioanalyzer. During extraction, 300 pg of a non-human synthetic miRNA (Arabidopsis thaliana ath-miR-159a) was added to each sample as a spike-in to monitor the technical variability during the isolation and for subsequent data normalization.

miRNA profiling was performed using TaqMan Low Density Arrays (TLDA) and pool A, which allows the analysis of expression profiles of 384 miRNAs. One nanogram of total RNA was reverse transcribed to cDNA, using TaqMan Advanced miRNA cDNA Synthesis Kit (Applied Biosystems, Thermo Scientific, Waltham, MA, USA), which is specific for the detection and quantification of mature human miRNAs in biological samples. The cDNAs were then amplified using the Universal miR-Amp Primers and Master Mix to uniformly increase the amount of cDNA for each target, maintaining the relative differential expression levels. The cDNA was loaded into the TaqMan Array Advanced miRNA array pool A and run in a 7900HT Fast PCR System (Applied Biosystems). miRNA data were analyzed with SDS RQ Software version 2.4 and with a Thermo Fisher Cloud app (Thermo Fisher Scientific, Waltham, MA, USA); miRNAs with Ct values ≥ 38 were considered as not expressed and excluded from further analysis [66].

Normalization was carried out by subtracting the hsa-miR-16 Ct from individual Ct values. The R-Bioconductor package Limma was adopted to evaluate the differential expression profile between the CEVs and AEVs.

Heatmaps were generated using GraphPad Prism^®^ release 6.0 software. Deregulated miRNAs were analyzed using the miRNet tool (https://www.mirnet.ca/miRNet/home.xhtml (accessed on 10 October 2023); pathway enrichment analysis was performed with the Function Explorer module (Database Reactome) and gene ontology was explored with the same module; the software uses standard enrichment analysis based on the hypergeometric tests after adjustment for false discovery rate.

miRNet integrates data from publicly available different miRNA databases such as TarBase, miRTarBase, and miRecords and allows users to construct miRNA-target interaction networks at different confidence levels.

### 4.15. Melanoma Cell Line Culture 

SK-MEL-28 cells, derived from primary malignant melanoma cells BRAF mutated, and SK-MEL-2, expressing mutant N-Ras (Q61R) (American Type Culture Collection, Manassas, VA, USA) were used and cultured in DMEM high glucose (Sigma Aldrich, St Louis, MO, USA) enriched with 10% fetal bovine serum (FBS), penicillin 100 U/mL, streptomycin 100 µg/mL, and 2 mM L-glutamine. The cells were maintained at 37 °C and 5% CO_2_.

### 4.16. Cell Cycle and Apoptosis Analyses

The quantitative measurements of the percentage of SK-MEL-28 in the different phases of the cell cycle were obtained through cytometric flow analyses by Muse^®^ technology (Luminex Corp., Austin, TX, USA) according to the manufacturer’s instructions. Cells were trypsinized and centrifuged, 72h after treatment with CM or EV from Amnio or Cesarean cells. The cells were fixed in 70% ethanol and frozen for at least 3 h prior to staining, after 2 washes in PBS, cells were resuspended in Muse^®^ cell cycle reagent and incubated at room temperature for 30 min. Finally, cells were run on the Muse Cell Analyzer.

The quantitative measurements of the percentage of SK-MEL-2 cells in the various stages of apoptosis or death were obtained through cytometric flow analyses by Muse^®^ technology (Luminex Corp., Austin, TX, USA), with Muse^®^ Caspase 3/7 kit according to the manufacturer’s instructions. Cells were trypsinized and centrifuged, 48h after treatment with CM or EV from Amnio or Cesarean cells. No fixed cells were stained with this no-wash, mix-and-read assay based on caspase 3/7 activity in combination with a cell death dye.

### 4.17. Migration Assay

Cell migration was measured by the wound-healing method, in melanoma cells [16]. Briefly, 2 × 10^5^ cells were seeded in 12-well culture plates. After 24 h, sterile P-100 pipette tips were used to make artificial perpendicular scratches on the cell layer, and the floating cells were washed twice with PBS. Appropriate complete medium, with CM or EV from Amnio or Cesarean cells, were replaced and, 36 h later, the distance of migration was photographed with an incubation monitory system, Provi CM20 (Olympus, Tokyo, Japan) at different time points and quantified with ImageJ software (US National Institutes of Health, Bethesda, MA, USA). 

### 4.18. Invasion Test

For the study of invasion capacity in 3D, 2-day spheroids were embedded in Matrigel for invasion assays. In brief, high proliferating SK-MEL-28 R1 cells [16] (3 × 10^3^) were induced to be spheroid by Cultrex 96 well 3D Spheroid BME cell invasion assay (R&D System, Minneapolis, MN, USA) and exposed to a conditioned medium of 3T3 cells as a chemoattractant. The images were taken with an EPView EP50 camera (Olympus, Tokyo, Japan) and quantified after 1 to 4 days using ImageJ-1.51 (NIH). 

### 4.19. Statistical Analysis

Experiments were performed in triplicate (biological replicates). For quantitative comparisons, values were reported as mean ± SD based on triplicate analysis for each sample. To test the significance of observed differences among the study groups One-way ANOVA with Bonferroni post hoc test or t student test were applied. A *p* value < 0.05 was considered to be statistically significant. Statistical analysis and plot layout were obtained by using GraphPad Prism^®^ release 6.0 software.

## Data Availability

The datasets presented in this article are not readily available because the data are part of an ongoing study. Requests to access the datasets should be directed to the corresponding author.

## Supplementary Materials

The following supporting information can be downloaded at: https://www.mdpi.com/article/10.3390/ijms252312502/s1.
