# Supplementary figures and images for: Evaluation of the Anti-Cancer Potential of Extracellular Vesicles Derived from Human Amniotic Fluid Stem Cells: Focus on Effective miRNAs in the Treatment of Melanoma Progression
Source: Int J Mol Sci. 2024 Nov 21;25(23):12502. doi: 10.3390/ijms252312502 (PMC11641077; doi:10.3390/ijms252312502)

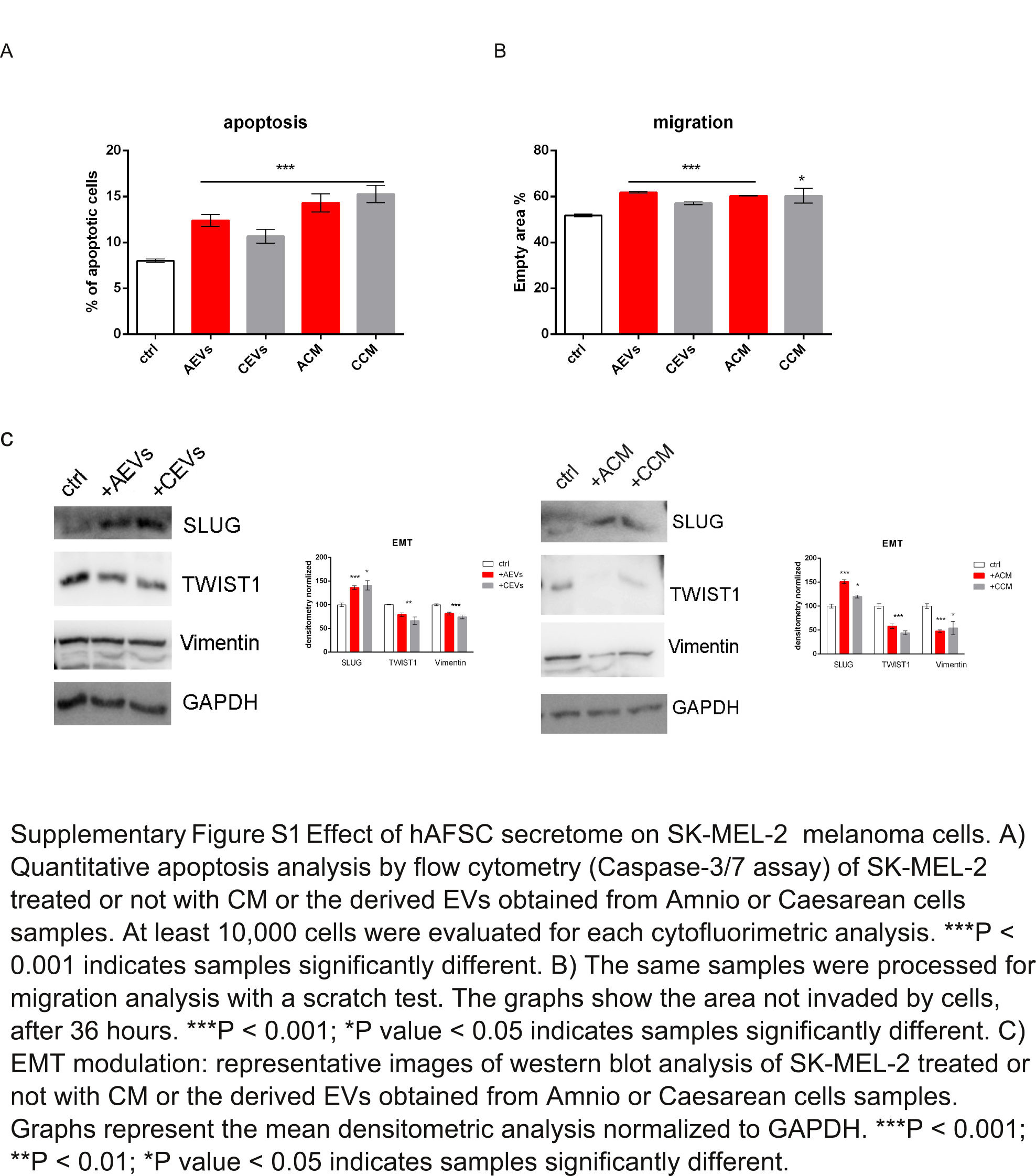

Supplement: Supplementary file 1 [file ijms-25-12502-s001.zip › ijms-3287081-supplementary.tif]
